# EBV and KSHV Infection Dysregulates Autophagy to Optimize Viral Replication, Prevent Immune Recognition and Promote Tumorigenesis

**DOI:** 10.3390/v10110599

**Published:** 2018-10-31

**Authors:** Mara Cirone

**Affiliations:** 1 Department of Experimental Medicine, “Sapienza” University of Rome, Viale Regina Elena 324, 00161 Rome, Italy; mara.cirone@uniroma1.it; Tel.: +39-06-49973319; Fax: +39-06-4456229; 2 Laboratory Affiliated to Istituto Pasteur Italia-Fondazione Cenci Bolognetti, 00161 Rome, Italy

**Keywords:** EBV, KSHV, autophagy, viral replication, cancer, DCs

## Abstract

Autophagy is a catabolic process strongly involved in the immune response, and its dysregulation contributes to the onset of several diseases including cancer. The human oncogenic gammaherpesviruses, Epstein—Barr virus (EBV) and Kaposi’s sarcoma-associated herpesvirus (KSHV), manipulate autophagy, either during the de novo infection or during the lytic reactivation, in naturally latently-infected lymphoma cells. In particular, the gammaherpesvirus infection reduces autophagy in immune cells, such as monocytes, resulting in the impairment of cell survival and cell differentiation into dendritic cells (DCs), which are essential for initiating and regulating the immune response. In the case of EBV, the reduction of autophagy in these cells, leading to p62 accumulation, activated the p62-NRF2-antioxidant response, reducing ROS, and further inhibiting autophagy. KSHV inhibits autophagy in monocytes by de-phosphorylating JNK2, altering the calpains–calpastatin balance and increasing the calpain activity responsible for the cleavage of ATG5. To further impair the immune response, KSHV also inhibits autophagy in differentiated DCs by hyper-phosphorylating STAT3. Conversely, when the lytic cycle is induced in vitro in latently-infected lymphoma B cells, both EBV and KSHV promote autophagy to enhance their replication, although the final autophagic steps are blocked through the down-regulation of Rab7. This strategy allows viruses to avoid the destructive environment of lysosomes, and to exploit the autophagic machinery for intracellular transportation. EBV and KSHV encode for proteins that may either inhibit or promote autophagy and, in addition, they can modulate the cellular pathways that control this process. In this review we will discuss the findings that indicate that autophagy is dysregulated by gammaherpesvirus to promote immune suppression, facilitate viral replication and contribute to the onset and maintenance of gammaherpesvirus-associated malignancies.

## 1. Introduction

The human gammaherpesviruses, Epstein–Barr virus (EBV) and Kaposi’s sarcoma-associated herpesvirus (KSHV), are DNA viruses strongly linked to hematologic diseases as well as several solid malignancies. EBV is a ubiquitous virus that infects more than 90% of the population worldwide, whereas KSHV infects a low percentage of the population, which varies depending on the different geographic areas. These viruses share several biologic properties, for instance, both reduce immune response, both infect B lymphocytes, and both establish latent or lytic infections, characterized by the expression of different sets of viral proteins [1,2]. Regarding EBV, three types of latency can be established: Latency I, in which only Epstein–Barr nuclear antigen (EBNA)-1, latent membrane protein (LMP)-2A/B, and EBERs are expressed; latency II, in which, in addition to latency I antigens, EBNA-2 or LMP-1 are expressed; and latency III, in which the expression of all EBNAs and LMPs can be detected [3]. During KSHV latency, a high expression of proteins, such as LANA, viral-Fas-associated death domain-like interleukin-1β (IL-1β)-converting enzyme-like inhibitory protein (v-FLIP), and v-cyclin, together with multiple microRNAs, and a low expression of K1, v-IL-6, and K15, can be detected [4]. In the course of the lytic phase, both viruses express the complete set of viral proteins, including those needed for virion assembling and enveloping. Shortly after infection, both EBV and KSHV initiate viral replication that is, in a short time, switched into latency. Most of the tumor cells naturally infected by gammaherpesviruses display a latent infection; however, viral replication can be induced in vitro upon exposure to opportune stimuli [5]. Interestingly, we have observed that such stimuli also activate autophagy that, in turn, promotes the viral replicative process [6,7].

Autophagy is one of the two main cellular catabolic pathways, which is finely regulated to maintain cellular homeostasis. Indeed, its dysregulation has been found to underlie a variety of diseases, including neurodegenerative diseases and cancer [8]. However, in the latter case, the role of autophagy is still controversial, as it may counteract the early phases of tumorigenesis while promoting the survival and progression of already established cancers [9]. Autophagy is articulated in several steps that go from autophagosome formation to their maturation and finally to fusion with the lysosomes, where the autophagic cargo is degraded together with the inner membrane of autophagosomes [10]. Each step is executed by several autophagy-related (ATG) proteins, under the control of cellular pathways such as PI3k/AKT/mTOR, MAPKs and STAT3. Autophagy is a selective and non-selective process that leads to the degradation of protein aggregates and damaged organelles such as mitochondria [11]. Autophagy plays an essential role in sustaining cell survival, particularly in conditions of stress, such as those induced by nutrient starvation or chemotherapies. Moreover, autophagy strongly contributes to the innate and specific immune response [12]. The majority of herpesviruses have developed strategies to inhibit autophagy, especially in immune cells, which is not surprising considering that this process is activated as a defensive response against microbial invasion. Autophagy indeed promotes pathogen elimination either directly, by redirecting microbes into the destructive environment of the lysosomes (xenophagy), or indirectly, contributing to MHC class II-mediated microbial antigen presentation [13]. The latter mechanism more efficiently occurs in the professional antigen presenting cells (APCs), such as dendritic cells (DCs) that are often susceptible to herpesvirus infection [14]. Among the herpesvirus proteins known to inhibit autophagy, there are those encoded by Herpes simplex (HSV) 1, such as ICP34.5 [15], and by Human cytomegalovirus (HCMV), such as TRS1 and IRS1 [14,16,17] that interact and inhibit Beclin-1, the molecule essential for the phagophore formation. However, autophagy can still have a pro-viral effect in the cases in which it is not blocked, as for example in the case of Varicella-zoster virus (VZV), in which the induction of autophagy sustains the survival of virus-infected cells [18] and promotes viral replication [19]. As it will be discussed later, autophagy is dysregulated by both EBV and KSHV, either to counteract the antiviral immune response or to promote viral replication and tumorigenesis.

## 2. EBV De Novo Infection and Autophagy

EBV mainly infects B lymphocytes and epithelial cells, although it has been reported to be able to infect other cell types too, including dendritic cells and their precursors, altering their immune function [20,21]. Dissimilar from the majority of proteins encoded by herpesviruses, several EBV proteins, especially those expressed during latency, seem to induce rather than inhibit autophagy. This is, for example, the case for LMP-1 that stimulates autophagy to promote its own degradation, regulating its turnover and maintaining the optimal expression level that prevents cytostasis and immune recognition [22]. It has been recently reported that LMP-2A may also promote the formation of autophagosomes and may up-regulate the expression of proteins involved in the autophagic pathway [23]. Among the other EBV-encoded latent nuclear proteins, a role in the positive regulation of autophagy has been recently reported also for EBNA-3C that, through this effect, inhibits apoptosis and sustains cell growth [24]. Interestingly, both EBNA-3C [25] and LMP-1 [26] play an essential role in the EBV-mediated B-cell transformation into lymphoblastoid cell lines (LCLs). In EBV-transformed LCLs, two different fractions of cells can be distinguished: The high- and low-proliferating fractions. The hyperproliferating fraction of LCLs needs a balanced level of autophagy to produce the biosynthetic intermediates necessary for cell growth, whereas the low-proliferating fraction of LCLs displays increased activation of AMPK, a reduced activation of mTOR, and a block of the autophagic flux at the final steps [27]. Autophagy inhibition could be mediated either directly, by EBV proteins, or indirectly, through the activation and inhibition of the cellular pathways involved in the regulation of autophagy. Although the majority of LCLs display a latency III program, a small fraction of them undergo spontaneous EBV replication [28]. It would be interesting to investigate whether autophagy is inhibited in the LCLs expressing EBV lytic proteins. This is because some of them, such as BamHI rightward fragment 1 (BHRF1) and BamH1 A leftward fragment 1 (BALF1), are orthologs of the cellular anti-apoptotic protein Bcl-2, which negatively regulates autophagy by binding to Beclin-1 and preventing the formation of the phagophore [29]. Interestingly, it has been reported that a mutant variant of the murine gammaherpesvirus γHV68, expressing a v-Bcl-2 variant not able to bind Beclin-1, is not able to establish a chronic infection in mice [30]. This indicates that the inhibition of autophagy mediated by v-Bcl-2 is of fundamental importance for viral persistence in the infected host. Moreover, since it is known that autophagy inhibition promotes the initial phases of cancerogenesis [31], it is possible that EBV could reduce autophagy to initiate the viral-driven tumorigenesis, through its encoded lytic proteins. Indeed, soon after infection, EBV replicates into the infected cells before establishing latency. Interestingly, we have shown that during viral replication induced in vitro by several different stimuli, the autophagic process is blocked at the final phases [6]. In line with these findings, our preliminary data suggest that the induction of autophagy at the beginning of EBV-mediated B-cell transformation may help to counteract LCL formation.

As reported above, autophagy plays an essential role in antiviral immune response, thus, EBV, like other viruses that persist in the infected host, must interfere with this process to avoid its own elimination. Besides the general contributions to the immune response, in the case of EBV, autophagy specifically promotes the presentation of EBNA1-derived peptides by MHC class [32]. Of note, EBNA1 does not promote the autophagic process, which differs from other EBV proteins expressed during the latency II or III [33].

Autophagy has been reported to contribute to the anti-EBV immune response mediated by plasmacytoid dendritic cells (pDCs), the main producers of type I IFNs. When these cells are infected by EBV, autophagy inhibition reduces the interaction between the virus and TLR7 and 9, located in the endosomal–lysosomal compartment of pDCs [34].

Even if the viral proteins involved remain to be identified, we have recently found that EBV infection efficiently inhibits autophagy and concomitantly reduces the intracellular ROS level in primary monocytes [35]. These effects could be responsible for the reduction of monocyte in-vitro differentiation into myeloid DCs previously observed [21]. Interestingly, autophagy and ROS have both been reported, in separate studies, to play an essential role in promoting the differentiating process of monocytes [36,37].

## 3. EBV Lytic Reactivation and Autophagy

The impact of autophagy on EBV lytic replication has been recently investigated by our and other’s laboratories. A study by Chien-Hui Hung et al. demonstrated that autophagy promotes EBV replication, and that the lytic protein Rta induces autophagy through the activation of ERK1/2 [38]. Accordingly, previous findings indicated that RTA was able to activate ERK1/2 [39] and that ERK1/2 promotes autophagy [40]. In line with these evidences, we demonstrated that autophagy was induced by the main treatments that induced the EBV lytic cycle, and confirmed that the inhibition of the first autophagic steps counteracted viral replication. In addition, we found that the final phases of autophagy were inhibited by EBV, allowing the virus to avoid its own elimination by the lysosomal proteases and to usurp the autophagic machinery for intracellular transportation [6]. Later on, we have demonstrated that, depending on the lytic cycle inducing treatment, a PKC theta–p38MAPK axis [41] or JNK1/2 [42] play a major role in promoting viral replication through the induction of autophagy. Other studies have investigated this aspect of EBV biology, demonstrating that the virus recruits Atg8–LC3 coupled membranes to its envelope in the cytosol, and uses autophagic membranes for efficient envelope acquisition during lytic infection [43]. Other authors have demonstrated that blocking the final steps of autophagy helps EBV replication, which may be due to a further inhibition of the final steps already induced by viral replication. However, by using different experimental approaches for the silencing of ATG proteins and different cell types, these authors have found that also inhibiting the initial autophagic steps could enhance EBV replication [44].

## 4. KSHV De Novo Infection and Autophagy

As reported for EBV, KSHV, the most recently discovered human gammaherpesvirus, can also infect several cell types, especially within immune cells. KSHV encodes for several proteins that mimic the cellular orthologs, a process known as molecular piracy. Among these proteins there are v-Bcl-2 and viral-Fas-associated death domain-like interleukin-1β (IL-1β)-converting enzyme-like inhibitory protein (v-FLIP), orthologs of c-Bcl-2, and c-FLIP, respectively. These proteins, besides preventing cell death, have a strong impact on the autophagic process. v-FLIP suppresses autophagy by preventing Atg3 from binding and processing LC3 [45], and is able to counteract the induction of autophagy mediated by another KSHV protein, v-cyclin (v-cyc) D [46]. The inhibition of autophagy by vFLIP in KSHV-infected endothelial long-term-infected telomerase-immortalized endothelial cells (TIVE-LTC), leads to the accumulation of p62/SQSTM-1, particularly in its Ser-403 phosphorylated form. This induces the degradation of Keap1 and the stabilization of Nrf2, and promotes angiogenesis and inflammation, and up-regulates the antioxidant response [47]. v-Bcl-2, besides cooperating with v-FLIP in inhibiting autophagy and counteracting apoptosis, has been recently reported to be essential for KSHV replication. However, this function correlates with an unusual localization of this protein within the cell nucleus [48]. As for EBV, we have found that KSHV infection inhibits autophagy in monocytes, resulting in a reduction of cell survival and in the impairment of DC formation. As a molecular mechanism to interfere with autophagy in differentiating monocytes, KSHV de-phosphorylates JNK2, alters the calpains–calpastatin balance, and increases the activity of calpains, which are responsible for ATG5 cleavage [49]. Moreover, KSHV is able to infect and reduce autophagy in already differentiated DCs, resulting in a dysregulation of their function. This occurs through the activation of STAT3, induced both directly, by viral infection, and indirectly, through the increased release of cytokines, such as IL-6 and IL-10, acting in an autocrine fashion [50,51]. Moreover, KSHV encodes for a viral protein, such as v-IL-6, that reinforces the effects of the cellular IL-6 and further phosphorylates STAT3 in the infected cells [50,52]. Although several KSHV proteins can negatively regulate autophagy, it should be considered that the expression of viral oncoproteins may induce the DNA damage response (DDR), which could activate the pro-autophagic functions of p53 by increasing the expression of its targets DRAM and Sestrin-1. In the case of KSHV, this mechanism has been demonstrated to be responsible for the induction of autophagy that is mediated by the viral protein v-cyclin [46]. However, it is also known that, through several strategies, including the activation of STAT3, gammaherpesviruses can repress the DDR to sustain cell proliferation [53]. Interestingly, in a recent study, we found an inverse correlation between p53 and STAT3 activation in KSHV-infected lymphoma cells, as the inhibition of STAT3 activates p53 [54]. Since we had previously found that the inhibition of STAT3 induces autophagy in KSHV-infected cells [51], it is possible that this effect could contribute to the activation of the pro-autophagic functions of p53. Interestingly, it seems that, depending on the subcellular localization, both p53 and STAT3 may play different roles in the regulation of autophagy [55,56]. A negative regulation of autophagy by p53 activation may be mediated by another KSHV protein, ORF57, that up-regulates the X-linked inhibitor of apoptosis (XIAP) [57]. The latter indeed contributes to autophagy inhibition through the degradation of Mdm2 and the stabilization of p53 [58]. Moreover, it is important to consider that KSHV-encoded proteins, such as LANA, inhibit p53 [59], repressing the transcription of its targets, likely including the pro-autophagic ones, during latency when LANA is not negatively regulated by its phosphorylation [59] or acetylation [60].

## 5. KSHV Lytic Reactivation and Autophagy

The first study that was aimed at investigating the impact of autophagy on KSHV replication was performed by Hui-Ju Wen et al. in 2010. The authors reported that the expression of the KSHV replication and transcription activator (RTA) induces autophagy that promotes the KSHV lytic cycle [61]. Autophagy was assessed based on the formation of LC3II, but the completeness of the autophagic flux, by the use of inhibitors of the lysosomal proteasis, was only assessed in 293T cells transfected with RTA, in a context in which the other KSHV proteins were not expressed. Later on, we confirmed the role of autophagy in promoting KSHV replication induced by TPA and butyrate combination (T/B), but we also showed that the last autophagic steps are blocked [7]. As in the case of EBV, this represents a smart strategy for KSHV to exploit autophagosomes as a means of intracellular transportation of viral particles. The down-regulation of RAB7 was found to be one of the underlying mechanisms leading to the autophagic block, as demonstrated by silencing this molecule [7]. Interestingly, the KSHV-encoded lytic protein, K7, has been reported to block autophagosome maturation by interacting with Rubicon [62]. K7 could contribute to the inhibition of the last autophagic steps that we observed during the activation of the KSHV lytic cycle by T/B. However, other KSHV proteins expressed during the lytic cycle, such as the v-G protein-coupled receptor (v-GPCR), could also negatively regulate autophagy, as it activates the PI3K/AKT/mTOR pathway, which is the master negative regulator of autophagy [63]. Interestingly, it has been reported that the autophagic protein Beclin-2 may influence the v-GPCR protein level, promoting its endo–lysosomal degradation [64]. It could be that, through the mTOR-mediated inhibition of autophagy, v-GPCR prevents its own degradation, promoting tumorigenesis, a process in which this viral protein plays an essential role [65]. Interestingly Sirolimus, which inhibits mTOR and activates autophagy, may delay KSHV-driven tumorigenesis [66]. Even if in this study the induction of autophagy and the delay of tumorigenesis were correlated with a reduced release of IL-6 and IL-10, it is possible that it could also depend on a reduction of the v-GPCR expression level. Furthermore, besides the activation of mTOR, v-GPCR could mimic the cellular homologue GPCR and reduce autophagy through the negative regulation of ATG14 L expression [67].

Regarding the first autophagic phases of autophagy that are activated by T/B and promote KSHV replication, they could be promoted by the KSHV latent lytic protein v-cyclin. This protein has reported to phosphorylate p53 at serine15 (Ser15-p53) and promote its pro-autophagic function [46]. Interestingly, we have recently reported that Ser15-p53 is activated by T/B and plays an important role in the induction of KSHV replication [42]. Therefore, it will be interesting to investigate whether p53 activation could promote the first autophagic phases in cells undergoing T/B treatment, through the expression of DRAM and/or Sestrin 1, given that both p53 and autophagy activation strongly promote KSHV lytic replication.

## 6. Conclusions

**Figure 1:**
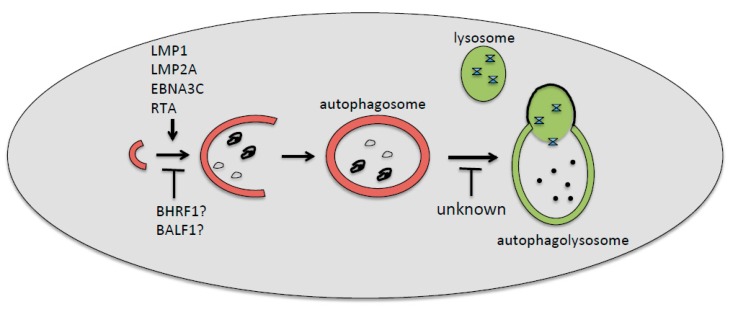
Epstein–Barr virus (EBV)-encoded proteins involved, or likely involved, in the positive or negative regulations of the first (LMP1, LMP2A, EBNA3C, RTA, BHRF1, and BALF1) or the last (unknown) autophagic steps. The inhibition of the first steps of autophagy by EBV has been found during de novo infection of immune cells, whereas the inhibition of the last degradative phases has been found mainly during the viral reactivation from latency.

**Figure 2:**
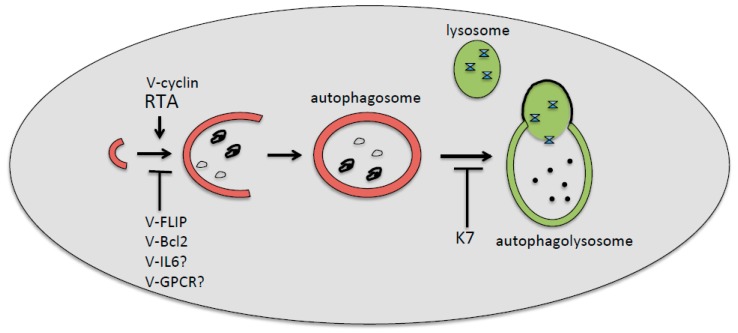
Kaposi’s sarcoma-associated herpesvirus (KSHV)-encoded proteins involved, or likely involved, in the positive or negative regulation of first (v-cyclin, Rta, v-FLIP, v-Bcl-2, v-IL-6, and v-GPCR) or the last (K7) autophagic steps. The inhibition of the first steps of autophagy by KSHV has been found during de novo infection of immune cells, whereas the inhibition of the last degradative phases has been found mainly during the viral reactivation from latency.

Although EBV and KSHV encode for several proteins that may inhibit or promote autophagy (Figure 1 and Figure 2), several studies from our and other groups suggest that the general outcome of viral de novo infection or viral reactivation from latency, in immune cells and cancer cells, respectively, is a reduction rather than activation of autophagy. This is not surprising given the pivotal role of autophagy in promoting the immune response and in counteracting the first phases of oncogenic transformation. In the latter case, autophagy helps cells to get rid of ROS and damaged organelles, whose accumulation contributes to cancer development. Thus, as in the case of cancerogenesis driven by the oncogenic gammaherpesviruses EBV and KSHV, autophagy could be inhibited to promote cellular transformation. Moreover, the induction of the first autophagic phases and the block of the last ones, occurring during gammaherpesvirus replication, may further contribute to the maintenance and progression of virus-associated cancers by enhancing viral production. Based on this knowledge, the induction of autophagy could hold the key to help counteract immune suppression, and to more successfully prevent or treat gammaherpesvirus-associated malignancies. The hypothesis that the induction of a complete autophagic flux could be a promising strategy against gammaherpesvirus-associated malignancies is further supported by considering the important role of autophagy in promoting the ATP release and the immune recognition of cancer cells, which are also essential for tumor eradication in the course of chemotherapies [68].
